# Isolated pancreatic tail hydatid cyst: A rare case report

**DOI:** 10.1016/j.radcr.2025.09.061

**Published:** 2025-10-16

**Authors:** Mahnaz Fosouli, Maryam Masjedi Esfahani

**Affiliations:** Imaging and Radiology Department, School of Medicine, Isfahan University of Medical Sciences, Isfahan, Iran

**Keywords:** Hydatid cyst, Pancreas, Pancreatic tail lesion

## Abstract

Hydatid disease is a zoonotic disease caused by a tapeworm, affecting the lung and liver. It is common in endemic areas, but often misdiagnosed. Ultrasound, computed tomography, and magnetic resonance imaging can reveal variable features, prompting alternative diagnoses. Hydatid cysts should be considered the first diagnosis for patients from endemic areas, even in organs not commonly affected. Here we report an extremely rare isolated pancreatic hydatid cyst affecting the tail and body; in imaging modalities, other lesions such as neoplasms and congenital cysts were initially considered.

## Introduction

Echinococcosis is a zoonotic disease caused by the Echinococcus tiny tapeworms and is classified as either cystic or alveolar forms. Hydatid disease is another name for cystic form, which is caused by the larval stage of Echinococcus granulosus [1].

It is found in the dogs and other carnivores as the definite hosts and the sheep, cattle, goat and pigs as the intermediate hosts. The humans are affected by food or water contaminated by dog feces, which contains the parasite eggs [2]. After ingesting the egg, the embryo releases and enters the portal venous system through the duodenal wall. These embryos have 3 possible outcomes: they can either die in the liver's capillaries, grow and induce hepatic hydatid cysts, or pass through the liver to implant in other organs, such as the lungs, where they may develop into hydatid cysts.

As this cycle shows, the lung and liver are the most commonly affected organs by hydatid disease. They can be rarely found in other organs as isolated cysts but are much more common with simultaneous involvement of the liver or lung or a history of previous involvement of those that had undergone surgery [3].

Primary or isolated pancreatic hydatid cysts are very rare even in the countries endemic for hydatid, and so they might be misdiagnosed as pancreatic neoplasms [4]. Pancreatic hydatid cysts account for less than 2% of hydatidosis, and even if they occur, they usually affect the pancreatic head rather than the body or tail due to better blood supply to the pancreatic head. This article aimed to report a case of an isolated pancreatic hydatid cyst affecting the body and tail, which is extremely rare.

## Case presentation

A 9-year-old girl presented to our hospital with cough and upper abdominal intermittent pain. Her drug and past surgical history were negative. She came from Semirom (a rural area of Isfahan Province), an endemic area for hydatid disease. She had no history of contact with dogs or cattle, sheep, and goats. Her family history revealed a recent hepatic hydatid cyst and PAIR procedure. Her physical examination was unremarkable.

Her hemoglobin level was 12.4 g/dL (reference value 11.5-15.5 g/dL), and her WBC count was 5200 mm-3 (reference value 5000-13000 mil/mm-3), with a normal absolute eosinophil count. Her liver and renal function, amylase and lipase tests were within normal limits.

**Fig. 1:**
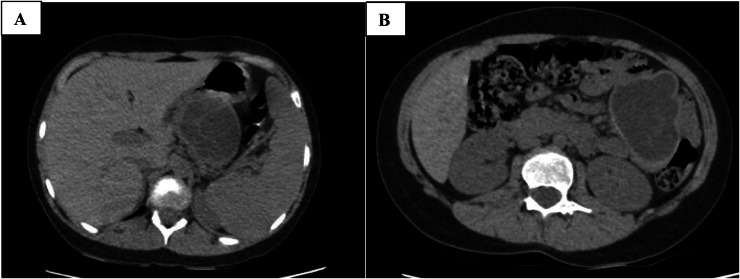
In limited view of abdomen of the high-resolution computed tomography images, a multiloculated cystic structure with hyperdense wall and septa is seen (A and B).

**Fig. 2:**
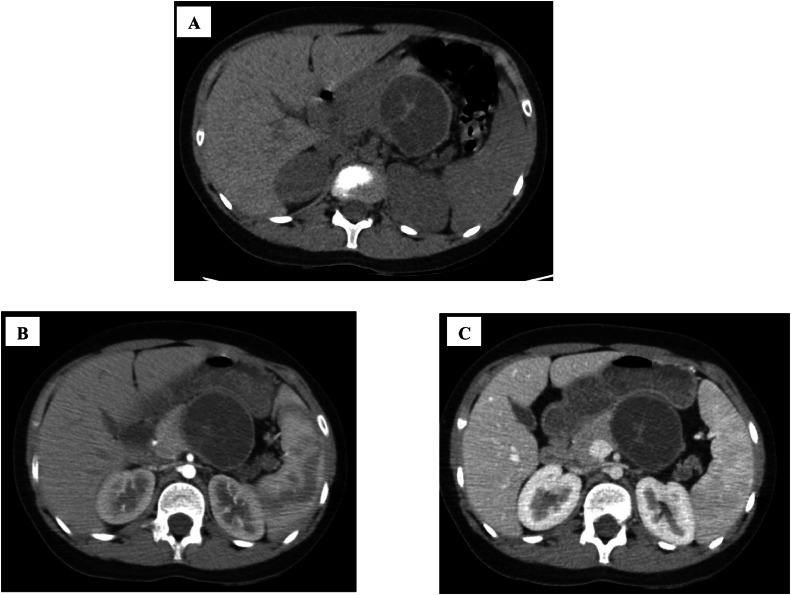
(A) Axial CT scan without contrast, (B) Axial contrast enhanced CT scan on arterial phase, and (C) on portal venous phase reveals a multi loculated fluid attenuated lesion (with average attenuation of 8 HU) in pancreatic body containing non enhancing relatively hyperdense septa.

**Fig. 3:**
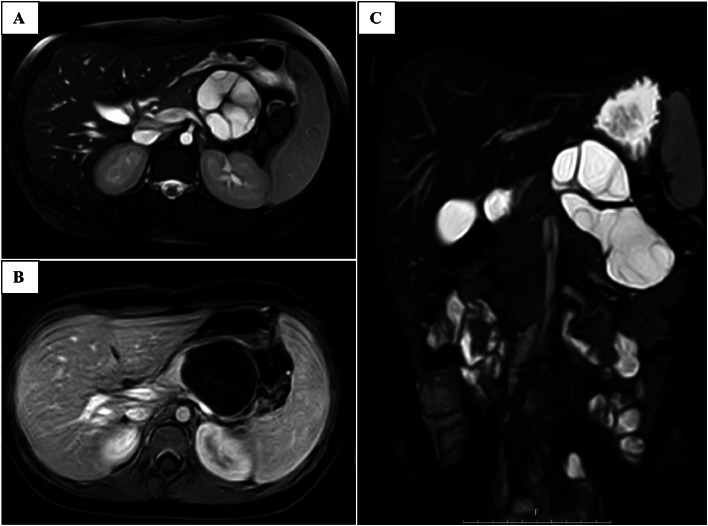
(A) Axial plane of fat saturated T2W MRI, (B) Axial plane of fat saturated T1W MRI with contrast, (C) Coronal plane of fat saturated T2 MRI. Images showed a multiloculated cystic lesion with benign appearance without solid component in the body and tail of pancreas. The lesion showed no enhancing septa or wall.

Due to the history of cough and to evaluate the possibility of pneumonia, high-resolution computed tomography (HRCT) of the lungs was performed. The lung fields were clear with no evidence of parenchymal infiltration or space-occupying lesions. But in the limited view of the upper abdomen a well-circumscribed, multiloculated cystic lesion with a relatively thick hyperdense wall and septa was demonstrated (Figs. 1,2, and 3).

In the abdominopelvic ultrasound, a multiloculated, thick-walled cystic lesion the size of 50*50*67 mm and with an approximate volume of 100 cc was seen in the pancreatic body and tail, with a very similar appearance to a type II hepatic hydatid cyst, so in combination with CT findings, the hydatid cyst was mentioned as the main diagnosis. She had no other lesions or abnormalities in her abdomen. Additionally, no gall stones were visible on ultrasound.

To check for cancerous growths, the patient had a contrast-enhanced CT of the abdomen and pelvis in the arterial and portal phases that included different types of imaging with and without contrast. To decrease radiation dose, CT without contrast was not done because the lesion was visible in the patient's HRCT. But unfortunately, the images were visualized without considering the pre-contrast images visible on HRTC and the hyperdense septa and wall were considered as enhancing and so congenital pancreatic cysts and less likely epithelial cysts (serous or mucinous), pseudocyst and remnants of mullerian cysts were reported. The hydatid cyst was also mentioned, but as a much less probable diagnosis according to its rarity and lab data correlation was recommended.

The patient underwent an MRI to solve this discordance, which showed a benign-appearing, multiloculated, thin-walled cystic lesion without a solid component was noted in the pancreatic body and tail. No evidence of true restriction diffusion was seen on DWI/ADC. Cystic lymphangioma (most favored), congenital cyst and, less likely, hydatid cyst was considered as differential diagnoses.

This patient finally underwent laparotomy. A large cyst was seen in the pancreatic body and tail, which resembled hydatid. A marsupialization procedure was done, and many daughter cysts were extracted. The surgical appearance was in favor of a hydatid cyst. But for further evaluation the cyst content and wall were sent for pathological investigation, which confirmed the diagnosis by visualization of Echinococcus granulosus and inflammatory cells. Post-surgery, our patient was administered albendazole (400 mg bi-daily for 1 month). The patient was discharged with stable vital signs and in satisfactory overall health.

## Discussion

Hydatid disease, caused by Echinococcus granulosus, is primarily found in the liver and lungs, with pancreatic involvement being rare. The patient's rural background raised suspicion due to higher transmission rates due to livestock farming and dog contact. However, the absence of direct exposure history and normal eosinophil count initially obscured the diagnosis.

Ultrasound (US) was used to diagnose a multiloculated cyst resembling a type II hepatic hydatid cyst, but the lack of saved images limited retrospective review. CT pitfalls led to overemphasis on neoplastic cysts or congenital cysts, emphasizing the importance of pre-contrast imaging. MRI discordance was also noted, with the thin-walled appearance being atypical for hydatidosis.

The diagnosis was missed initially due to the rarity of pancreatic involvement, overreliance on lab findings, and imaging mimickers. Intraoperative findings showed the visualization of daughter cysts during laparotomy as pathognomonic for hydatid disease, and marsupialization was appropriate for uncomplicated cysts. Histopathology confirmed the diagnosis with the presence of the E. granulosus laminated membrane and surrounding granulomatous inflammation.

Key lessons for clinicians include considering hydatid disease in endemic areas, using multimodal imaging, recognizing the role of serology, and avoiding radical resections for uncomplicated cysts. Future considerations include molecular diagnostics like polymerase chain reaction (PCR) of cyst fluid for rapid confirmation [5], and long-term monitoring with imaging to detect recurrence in approximately 10% of cases. ELISA is a reliable method for diagnosing hydatid cyst infection, with high sensitivity and specificity ranging from 80% to 97%. Indirect Hemagglutination (IHA) and Indirect Fluorescent Antibody (IFA) are used to confirm the presence of antibodies in ELISA-positive samples [6].

Hydatid cysts can be detected using sophisticated cross-sectional imaging, contrast-enhanced methods, diffusion-weighted imaging, Doppler ultrasonography, serological and molecular correlation, along with heightened awareness and training among radiologists. These approaches assist in distinguishing hydatid cysts from neoplastic lesions, evaluating vascularity and wall enhancement, and differentiating hydatid cysts from neoplastic lesions. Additionally, while both pancreatitis related pseudocyst and pancreatic hydatid cyst can appear cystic, hydatid cysts may have calcified walls and daughter cysts, whereas pseudocysts typically have a more inflammatory appearance with surrounding fat stranding. Hydatid cysts are typically cystic lesions with well-defined margins and may contain daughter cysts. Solid pseudopapillary tumors, on the other hand, are well-encapsulated tumors with a mix of enhancing solid and cystic components due to hemorrhage and degeneration.

This case highlights the diagnostic challenges of pancreatic hydatid cysts, a rare manifestation of Echinococcus granulosus infection, particularly in non-endemic regions. The patient's rural origin in Semirom, Iran, made the diagnosis complicated due to the absence of direct animal contact and normal eosinophil count.

Hydatid cysts typically exhibit pathognomonic imaging features, such as a thick wall, septations, and daughter cysts on ultrasound, CT, or MRI [7]. However, in this case, contrast-enhanced CT misinterpreted the hyperdense septa as enhancement, favoring congenital or neoplastic cysts [8]. MRI prioritized cystic lymphangioma due to the lesion's multiloculated, thin-walled appearance [9]. Definitive diagnosis was achieved through laparotomy, with histopathology confirming E. granulosus. Marsupialization, a conservative surgical approach, was effective, aligning with recommendations for uncomplicated cysts.

## Conclusion

Although rare, isolated pancreatic hydatid cysts should be considered as an important diagnosis for patients from endemic areas if US appearance is suggestive. We should also be careful about enhancement on CT, as it determines our differential diagnosis, and we should compare before and after contrast images to define enhancement.

## Patient consent

The written, informed consent for publication of their case was obtained from the patient.
